# AuReTim: an inexpensive and extensible open-source auditory psychomotor vigilance test

**DOI:** 10.3389/frsle.2023.1168209

**Published:** 2023-07-21

**Authors:** Torsten Straßer, Inga Rothert, Thomas Heine, Tobias Peters

**Affiliations:** ^1^Institute for Ophthalmic Research, Centre for Ophthalmology, University of Tübingen, Tübingen, Germany; ^2^University Eye Hospital, Centre for Ophthalmology, University of Tübingen, Tübingen, Germany; ^3^STZ eyetrial at the Centre for Ophthalmology, University of Tübingen, Tübingen, Germany; ^4^Institute for Energy and Automation Technology, Lighting, Technical University Berlin, Berlin, Germany; ^5^Institute of Physical and Theoretical Chemistry, University of Tübingen, Tübingen, Germany

**Keywords:** psychomotor vigilance, open-source, portable PVT, Java, Raspberry Pi, sleep research, attention

## Abstract

Within a large joint research project aiming for characterizing the nonvisual effects of light (NiviL), AuReTim, a low-cost and extensible open-source portable psychomotor vigilance test using auditory stimuli was developed, tailored for field testing. Currently, an unprepared simple reaction time and a go/no-go paradigm using acoustic stimuli are implemented. AuReTim is based on inexpensive hardware, e.g., its core is a Raspberry Pi leveraging a touch screen as input. Its software is developed in Java™ using open-source libraries, therefore providing connectivity with other research setups, e.g., EEG, and easy extensibility with other stimulus paradigms. A simulation study proved the precise timing of AuReTim with limits of agreement between −1.86 and 1.67 ms. AuReTim combines the mobility of tablet-based psychomotor vigilance tests with the usability of conventional computer-based tests, which is especially helpful in field studies. AuReTim was successfully applied to study the effects of different lighting on alertness and proved to be a valuable tool for studies using the central nervous activation level as an outcome measure.

## 1. Introduction

The non-visual effects of light have gained a special interest in recent years and the large joint research project NiviL, funded by the German Federal Ministry of Education and Research (13N13524, 13N13398) and involving diverse disciplines, was initiated to investigate the non-visual effects of short-wavelength light on circadian rhythms, alertness, and wellbeing in humans. The investigations covered people of different ages in different life situations, as well as during illness and stress at different times of the day and over the seasons of the year (Völker, 2018; Tübingen, 2019; Petrowski et al., 2020).

The effects of light on the circadian rhythm and alertness can be assessed with the Psychomotor Vigilance Test (PVT) (Chang et al., 2013), a neurocognitive assay for sleep loss (Dorrian et al., 2004) and among the most sensitive tests for sleep restriction and the most practical for use in an operational environment (Balkin et al., 2004).

One of the sub-projects of NiviL focused on the effect of light on residents of retirement homes. For this, we developed a simple-to-use, low-cost, and portable psychomotor vigilance test using auditory stimuli, AuReTim. AuReTim is based on the single-board computer Raspberry Pi and open-source software. Here we present detailed information about the building blocks of AuReTim, the features it implements currently as well as data on the timing accuracy of AuReTim.

## 2. Method

### 2.1. Hardware

**Figure 1:**
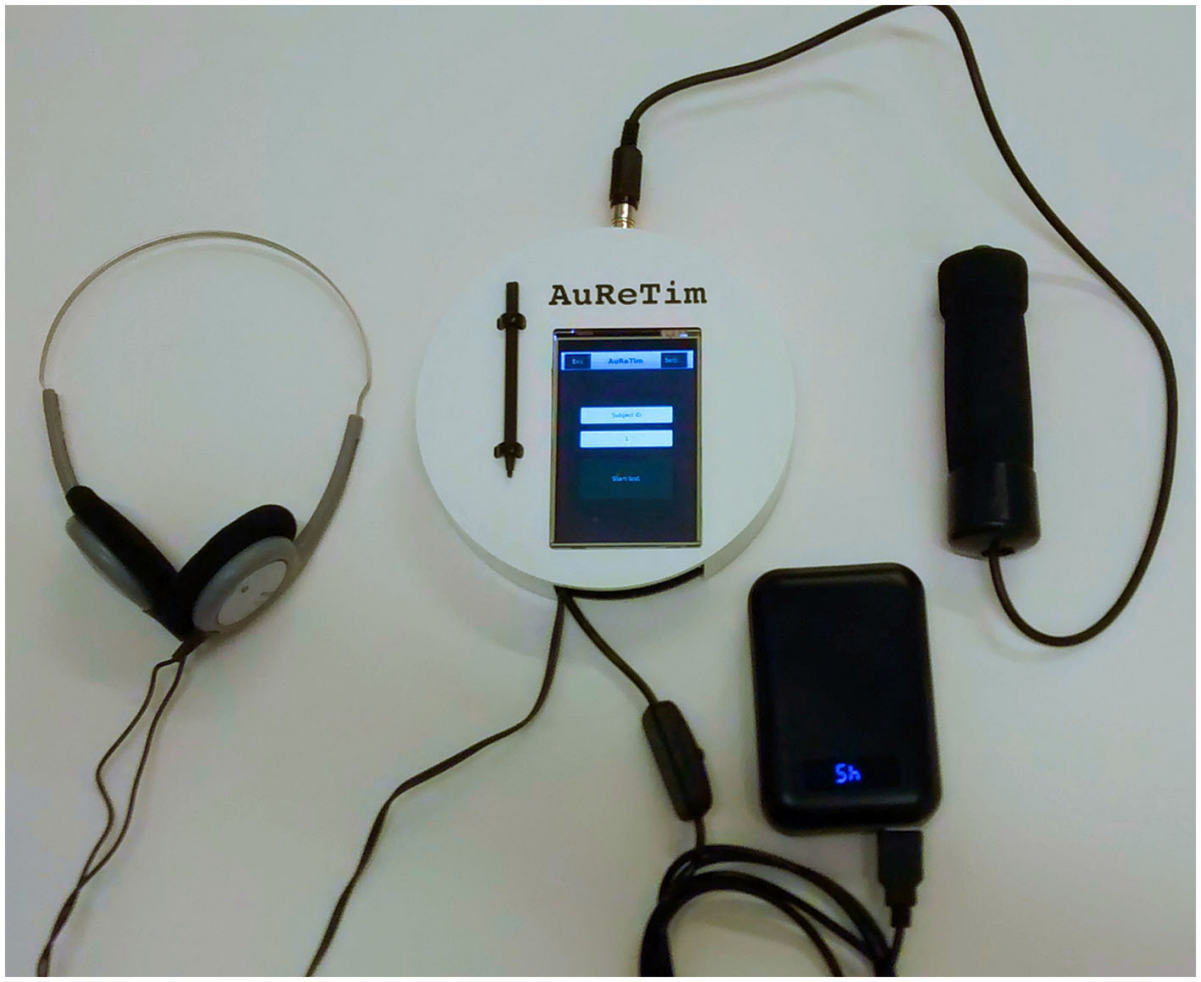
Photograph of the AuReTim psychomotor vigilance test with the device itself with the touchscreen and an attached touchscreen pen in the center, headphones at the left, hand-held push button at the right, and the switchable battery pack for portable usage at the lower right of the device.

Figure 1 shows the parts of the AuReTim psychomotor vigilance test. The core of AuReTim is the single-board computer Raspberry Pi Model B (Raspberry Pi Foundation, Caldecote, UK), however, any single-board computer that provides support for Java (version ≥8) should be suitable. A 3.5″ resistive touchscreen with a resolution of 320 × 480 pixels (LCD(A), Waveshare Electronics, Shenzhen, China) attached to the General Purpose Input/Output (GPIO) ports and controlled using the Serial Peripheral Interface (SPI) of the Raspberry Pi provides the user interface for input and output of the AuReTim software. The acoustic stimulus is generated using the internal soundcard of the Raspberry Pi and delivered by commercially available headphones (Philips SBCHL140) connected to the audio jack. The participant's response to the acoustic stimulus is captured using a hand-held push-button (Novel Electronic Designs, Inc. Chilicothe, Illinois, USA) attached to the GPIO ports. Power is provided by a rechargeable battery pack (Model X2, Li-ion, 10,000 mAh, 5 V/2.1 A, iMuto, South El Monte, CA, USA). A commercially available case (Orbital Case, Polypodis UG, Berlin, Germany) was altered to accommodate the touch display and the connector for the push button. A complete list of all parts used for an AuReTim system with manufacturers and prices is given in Table 1. The total cost for the system is less than € 150 (as of December 2020).

**Table 1:** List of parts used for an AuReTim system including manufacturers and prices.

	**Type**	**Manufacturer**	Price^*^
Raspberry Pi	Model 3B	Raspberry Pi (Trading) Ltd.	€ 35
Touchscreen	3.5″ RPi LCD (A) 320^*^480 IPS TFT Touch Screen	Waveshare Electronics	€ 20
Earphones	SBCHL140	Philips GmbH	€ 10
Button	Hand-held button	Novel Electronic Designs Inc.	€ 13
Case	Orbital Case	Polypodis UG	€ 16
Powerbank	X2, 10000 mAh, 5 V/2.1 A	iMuto	€ 30
SD card	32 GB microSDHC Class 10	Intenso	€ 7
Switchable cable	K-1470, USB A to Micro-B	Renkforce	€ 6
Cables, misc.			€ 5

The total cost for an AuReTim system is less than € 150.

* Estimated prices as of December 2021.

### 2.2. Software

AuReTim leverages Raspbian, a port of Debian Wheezy optimized for the ARM-based Raspberry Pi, as the operating system. The AuReTim program is developed in Java and JavaFX and is executed in the Oracle^®^ Java 8 runtime environment (Oracle^®^ Java 8, hard-float ABI ARMv7, Oracle Corp., Redwood City, CA, USA), running on top of Raspbian. As an open-source alternative to Oracle^®^ Java, Liberica Java (BellSoft Ltd., San Jose, CA, USA) may be used.

The AuReTim code utilizes several open-source libraries, listed in Table 2: ControlsFX for high-quality user-interface controls and Flatter as a modern JavaFX theme specialized for touch-based applications, Pi4J for an object-oriented application programming interface to the full I/O capabilities of the Raspberry Pi in Java, and Apache Commons Math and Apache Commons CSV for the statistical calculations and text export of results as character separated values formatted files. The build process and the installation of the AuReTim software are described in Supplementary material 1.

**Table 2:** Open-source libraries used by AuReTim.

**Library**	**Version**	**License**	**Homepage**
ControlsFX	8.40.10	BSD-3-Clause	https://github.com/controlsfx/controlsfx
Flatter	0.7	BSD-3-Clause	http://www.guigarage.com/flatter
Pi4J	1.0	LGPL-3.0	https://pi4j.com
Apache Commons Math	3.6	Apache-2.0	https://commons.apache.org/proper/commons-math
Apache Commons CSV	1.2	Apache-2.0	https://commons.apache.org/proper/commons-csv

### 2.3. Determination of the temporal accuracy

To determine the accuracy of the stimulus and the recorded response delay of AuReTim, the generated audio signal, and the button response was registered along with a trigger impulse issued using a Raspberry Pi GPIO pin at the start of a test sequence using the analog inputs of an OpenBCI bio-sensing device (OpenBCI Cyton Board, OpenBCI, New York, USA) with a sampling frequency of 250 Hz. The button presses were simulated by toggling another Raspberry Pi GPIO pin, thereby short-circuiting the input line of the hand-held button. The unprepared simple reaction test paradigm with a 500 ms audio stimulus and 1,000 repetitions was used to record the response delays, whereby the delays of the simulated user responses varied between 300 and 990 ms in steps of 10 ms after sequence onset. The simulated response delay was assessed using the OpenBCI device, referred to as the “measured delay.” This measurement was then compared to the recorded response delay obtained from the AuReTim software, termed the “recorded delay,” as well as the precise delay derived from the simulated button presses, known as the “expected delay.” Moreover, the exact duration of the audio stimulus was also measured.

The main criteria of the AuReTim test evaluation were proof of a linear relationship between recorded and expected response delays, the quantification of possible systematic errors, and the evaluation if the system's inherent delays are within the limits as suggested by Basner et al. (2021).

In order to mitigate the systematic error introduced by the sampling of 4 ms intervals using the OpenBCI device, the mean values and variances of the measured timing data were adjusted by incorporating the expected value (Equation 1) and the variance (Equation 2) of the systematic error (se), assuming a discrete uniform distribution for the time delays.


(1) $$\begin{array}{lll} E\left(se\right) & = & \frac{1}{n}\overset{n}{\underset{i=1}{\sum }}se_{i};se\in \left\{0,\;1,\;2,\;3\right\}\;ms \end{array}$$

(2) $$\begin{array}{lll} VAR\left(se\right) & = & \frac{1}{n}\overset{n}{\underset{i=1}{\sum }}{\left(se_{i}-E\left(se\right)\right)}^{2} \end{array}$$

By virtue of Equation 3, which establishes the mean measured delay as the combined effect of the mean true delay, the expected value of the systematic error, and the mean random error originating from both the device and the software, it becomes possible to estimate a corrected delay, excluding the systematic error, through the utilization of Equation 4.


(3) $$x_{measured}\;=x_{delay}+E\left(se\right)+\varepsilon_{random}$$

(4) $$x_{corrected}\;=x_{measured}-E\left(se\right)$$

Based on the property that the variance of a sum of uncorrelated random variables is equal to the sum of their individual variances, an analogous approach can be employed to estimate a corrected variance (Equation 5).


(5) $$\begin{array}{l} VAR\left(x_{corrected}\right)=VAR\left(x_{measured}\right)-VAR\left(se\right) \end{array}$$

Measured and corrected data were analyzed using Bland-Altman's method (Bland and Altman, 1986) and *t*-tests. Additionally, linear regression analyses were performed to investigate a potential proportional relationship between the means and differences of the corresponding sets of measured, recorded, and expected data. All analyses were performed using SAS JMP^®^ (Version 15.2.1. SAS Institute Inc., Cary, NC, 1989-2019).

## 3. Results

### 3.1. Auretim features and functionality

**Figure 2:**
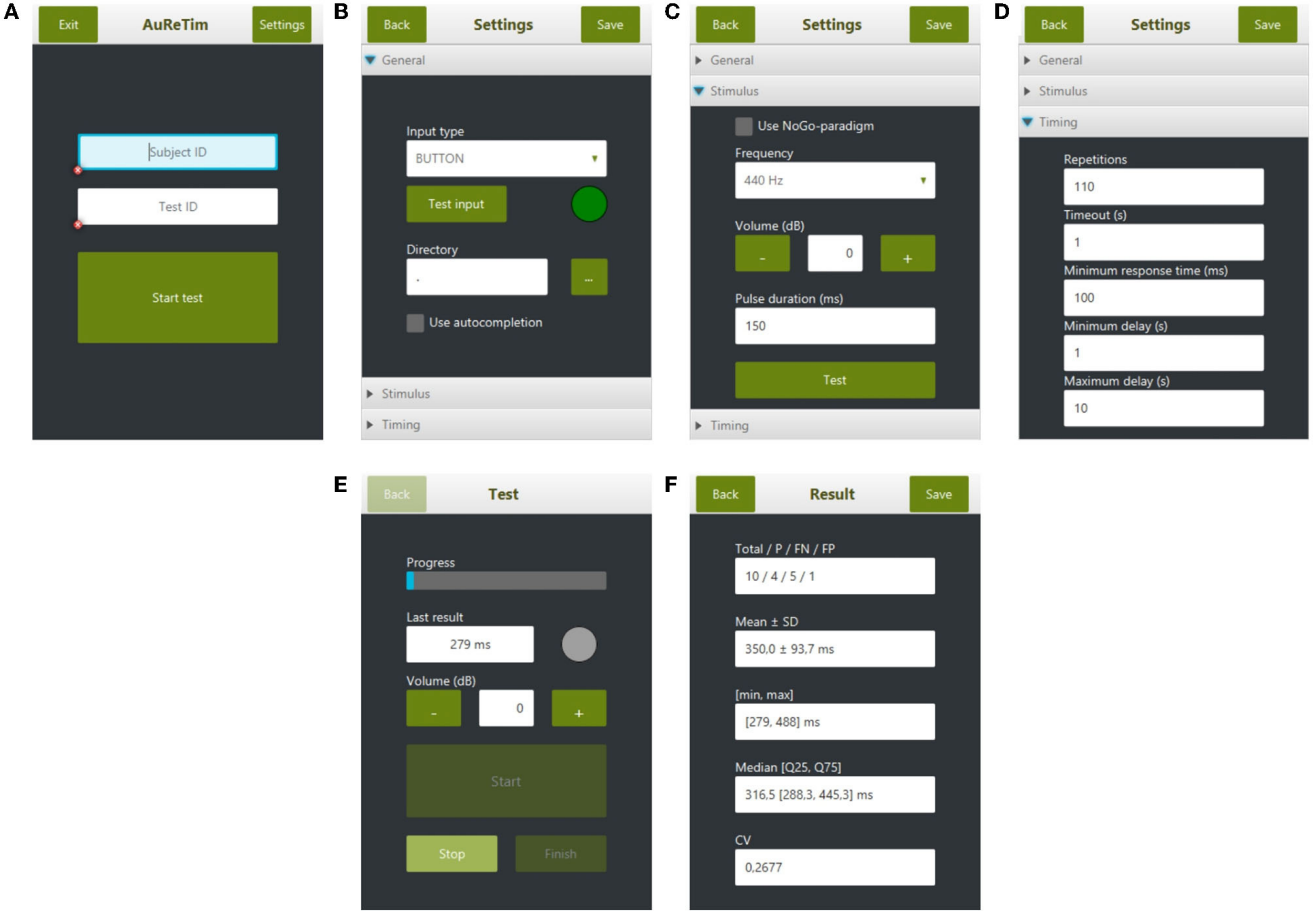
Overview of the user interface of AuReTim: **(A)** start screen with input fields for subject and test id, **(B)** general settings for input type and directory selector for saved results, **(C)** stimulus settings for selecting the stimulus paradigm and the configuration of the auditory stimuli, **(D)** timing settings for the stimulus paradigm, **(E)** test screen shown during the test with the last response time and the progress, **(F)** results of a test including the number of total, true-positive, false-negative, and false-positive responses as well as descriptive statistics.

AuReTim currently implements two paradigms for psychomotor vigilance: an unprepared simple reaction time test (Wilkinson and Houghton, 1982; Dinges and Powell, 1985; Dorrian et al., 2004), testing the tonic activation of the central nervous system (Weess et al., 2000), and a go/no-go paradigm (Donders, 1969) for testing selective attention (Weess et al., 2000). The type of input (button, keyboard, mouse) is defined in the AuReTim settings (Figure 2B).

The auditory stimulus can be configured for frequency, duration, and volume. By default, a tone with a frequency of 440 Hz (A4, pitch standard) and a duration of 150 ms is preset. If the go/no-go paradigm is selected (Figure 2C), two tones are used, whereby the tone for no-go is the quadruple of the selected tone (i.e., go = 440 Hz, no-go = 1,760 Hz) and both tones use the same duration (Figure 2C).

In the simple reaction time test, the inter-stimulus interval for the auditory stimuli is defined using a minimum and a maximum delay from which the actual delay is selected randomly during the test. To set up a test according to the original used by Wilkinson and Houghton (1982), a minimum and maximum delay of 1 and 10 s, respectively should be configured with 110 repetitions for a test duration of about 10 min (assuming a mean inter-stimulus interval of 4.5 s). In the go/no-go paradigm, a fixed delay is used, according to the set minimum delay (Figure 2D). Additionally, a minimum response time can be defined: responses faster than this value are considered false positives.

During the test, the current progress and the last response time are displayed (Figure 2E). A running test may be interrupted and continued at a later time point until the number of required repetitions is reached. At the end of the test, the results including the number of total, true-positive, false-negative, and false-positive responses, as well as descriptive statistics (minimum and maximum response time, mean and standard deviation, and median and quartiles of the response time) are displayed (Figure 2F). The results can be saved as character-separated values text files in a directory (e.g., on a USB key) specified in the settings (Figure 2B) using a filename generated from the subject id and the test id (Figure 2A). The saved files contain all single responses of a participant with their exact response time, classified as correct, false negative, or false positive to be used for further analysis (Verbruggen and Logan, 2008).

The developed AuReTim system was certified by a Notified Body (TÜV-Süd Group, IS-EG1-09.03.16-1) as a Class IIa medical device according to the European guideline for medical devices MDD 93/42/EEC.

### 3.2. Auretim timing accuracy

**Figure 3:**
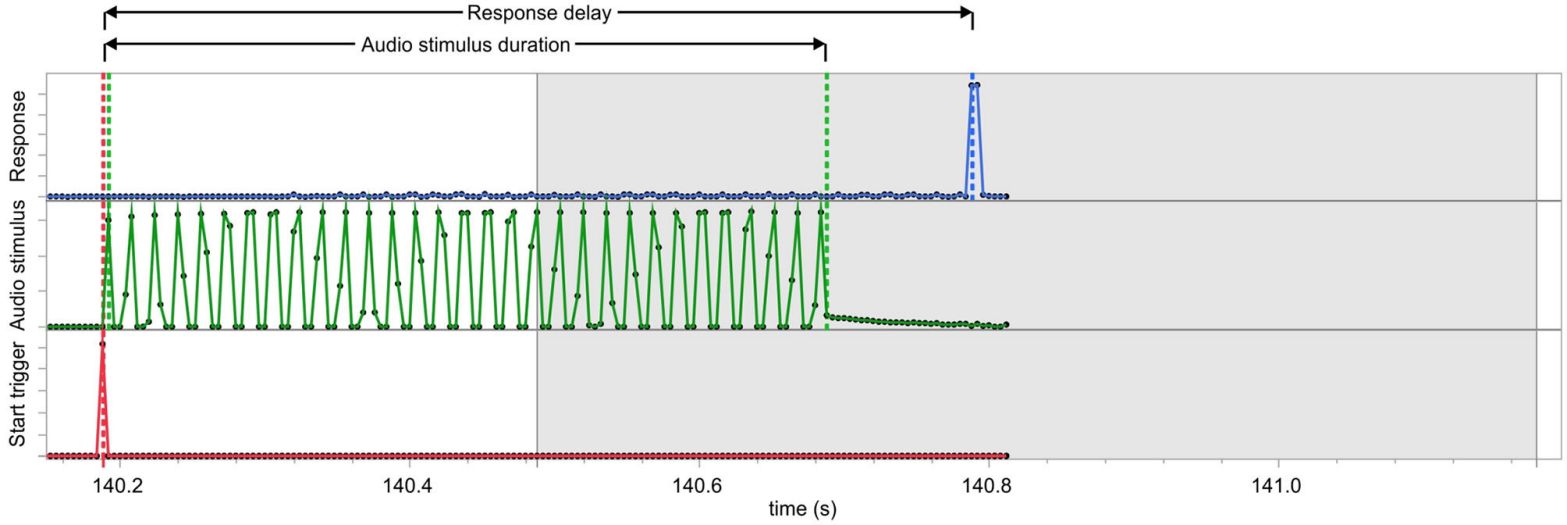
Excerpt of the recording using the analog input of the OpenBCI system used to measure the timing of the AuReTim device. The lower trace (red) shows the trigger signal sent by the software when a sequence is started; the middle trace (green) depicts the audio stimulus recorded from the audio output of the Raspberry Pi; the upper trace (blue) shows the signal of the simulated button press; the gray shaded area represents the time frame (300–990 ms, in 10 ms steps) within the button response is simulated. The response delay is calculated as the time duration between the start trigger and the response signal onset.

Figure 3 depicts the determination of the time duration at an excerpt of the recording of the generated audio tone, the trigger signals, and the simulated button press performed with the Open BCI system. For each trial, a button press is simulated with an increasing time delay in steps of 10 ms between 300 and 900 ms after stimulus onset.

The audio stimulus duration followed a normal distribution with a mean and standard deviation of 504.3 ± 4.9 ms. The minimum and maximum stimulus duration were 488 ms and 516 ms, respectively. A one-sample *t*-test revealed a statistically significant prolongation of 4.3 ms compared to the expected audio duration of 500 ms [*t*_(999)_ = 27.4778, *p* < 0.0001].

**Figure 4:**
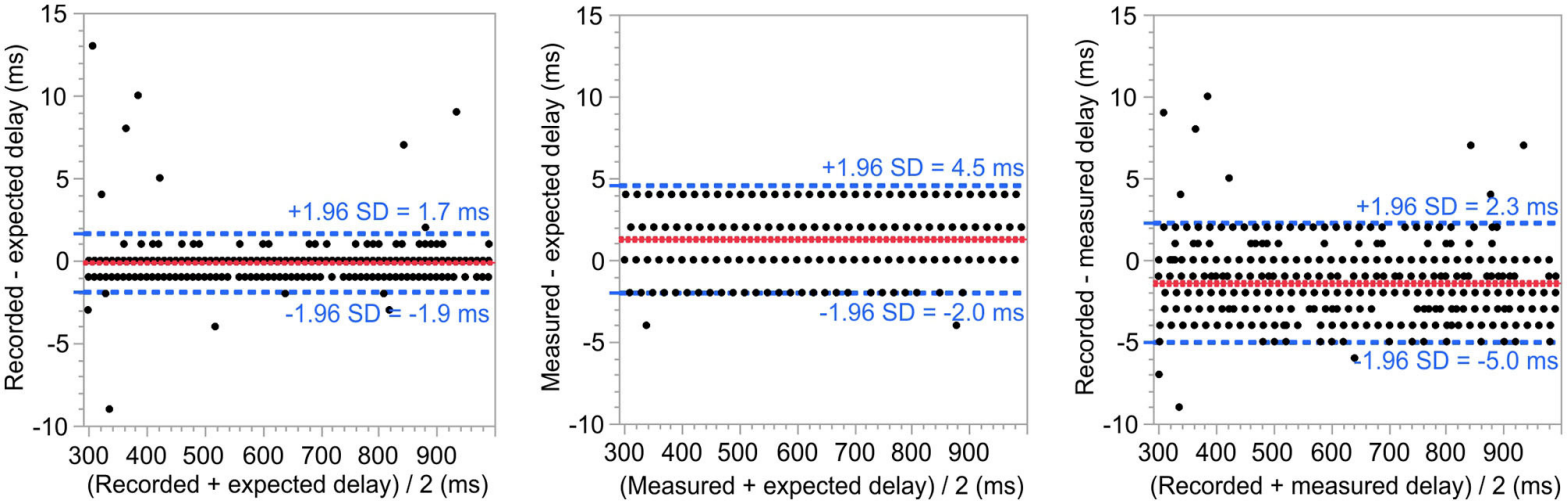
Bland-Altman plots comparing the expected response delay with the recorded response delay of the AuReTim software **(left)** and the response delay measured using the OpenBCI system **(center)**, respectively, as well as the recorded and the measured response delay **(right)**. Mean differences are depicted as solid red, limits of agreement as dashed blue lines. None of the plots shows a systematic bias. Additionally, the plots of the measured responses **(center, right)** indicate, that a large part of the jitter is most likely caused by the time resolution (4 ms) of the OpenBCI system.

The analysis of the response delays reported by the AuReTim software and measured using the OpenBCI system using Bland-Altman plots showed no systematic biases concerning the expected delays of the simulated button presses (Figure 4, left chart). The comparisons with the measured responses reveal the presence of a systematic error, which arises from the sample rate of the OpenBCI system (Figure 4, middle and right chart).

The differences between all the compared time durations were visually inspected for following a normal distribution. A paired-sample *t*-test revealed a statistically significant mean difference of 0.1 ms between the expected response delay and the one recorded by the AuReTim software with rather narrow limits of agreement (−1.86 to 1.67 ms). The comparisons between measured vs. expected and recorded vs. measured unveiled statistically significant differences of greater magnitude (1.39 ms, 1.30 ms), with wider limits of agreement (−5.03 to 0.11 ms, −2.39 to 1.99 ms). The comparisons involving the corrected measurements, which account for the estimated systematic error of the OpenBCI system, reveal statistically significant differences within a range similar to those observed between the recorded and expected value (−2.89 ms, −0.20 ms; Table 3).

**Table 3:** Results of paired-samples *t*-tests comparing the expected time delays of the simulated button presses with those determined by the AuReTim software and measured using the OpenBCI system.

**Response delay (*n* = 1,000)**	**Diff. mean ±SEM (ms)**	***t*-statistics**	***p*-value**	**Limits of agreement (ms)**
Recorded vs. expected	−0.10 ± 0.03	*t*_(999)_ = −3.4032	0.0007	(−1.86, 1.67)
Measured vs. expected	1.30 ± 0.05	*t*_(999)_ = 24.7628	< 0.0001	(−1.95, 4.54)
*Corrected vs. expected*	–*0.20 ± 0.04*	*t_(999)_ =* –*3.6569*	* < 0.0001*	*(*–*2.39, 1.99)*
Recorded vs. measured	−1.39 ± 0.06	*t*_(999)_ = −23.7229	< 0.0001	(−5.03, 2.25)
*Recorded vs. corrected*	–*2.89 ± 0.05*	*t_(999)_ =* –*59.6161*	* < 0.0001*	*(*–*5.89, 0.11)*

Values in italics have been adjusted to account for the systematic error arising from the sampling rate of the OpenBCI system.

The results of the linear regression analyses revealed statistically significant slopes for the measured and expected response delay [slope = 0.0017, 95% CI: (0.0012, 0.0022), *t*_(998)_ = 6.66, *p* < 0.0001] and for the recorded and measured response delay [slope = −0.0016, 95% CI: (−0.0021, −0.0010), *t*_(998)_ = −5.46, *p* < 0.0001]. However, no statistically significant slope was found for the recorded and expected response delay [slope = 0.0001, 95% CI: (−0.0002, 0.0004), *t*_(998)_ = 0.87, *p* = 0.3831]. Notably, a statistically significant intercept was only observed for the recorded and measured response delay [intercept = −0.3972 ms, 95% CI: (−0.7726, −0.0219) ms, *t*_(998)_ = −2.08, *p* = 0.0381].

The raw data of the simulation experiment can be found in Supplementary material 2.

### 3.3. Example use: NiviL study

**Figure 5:**
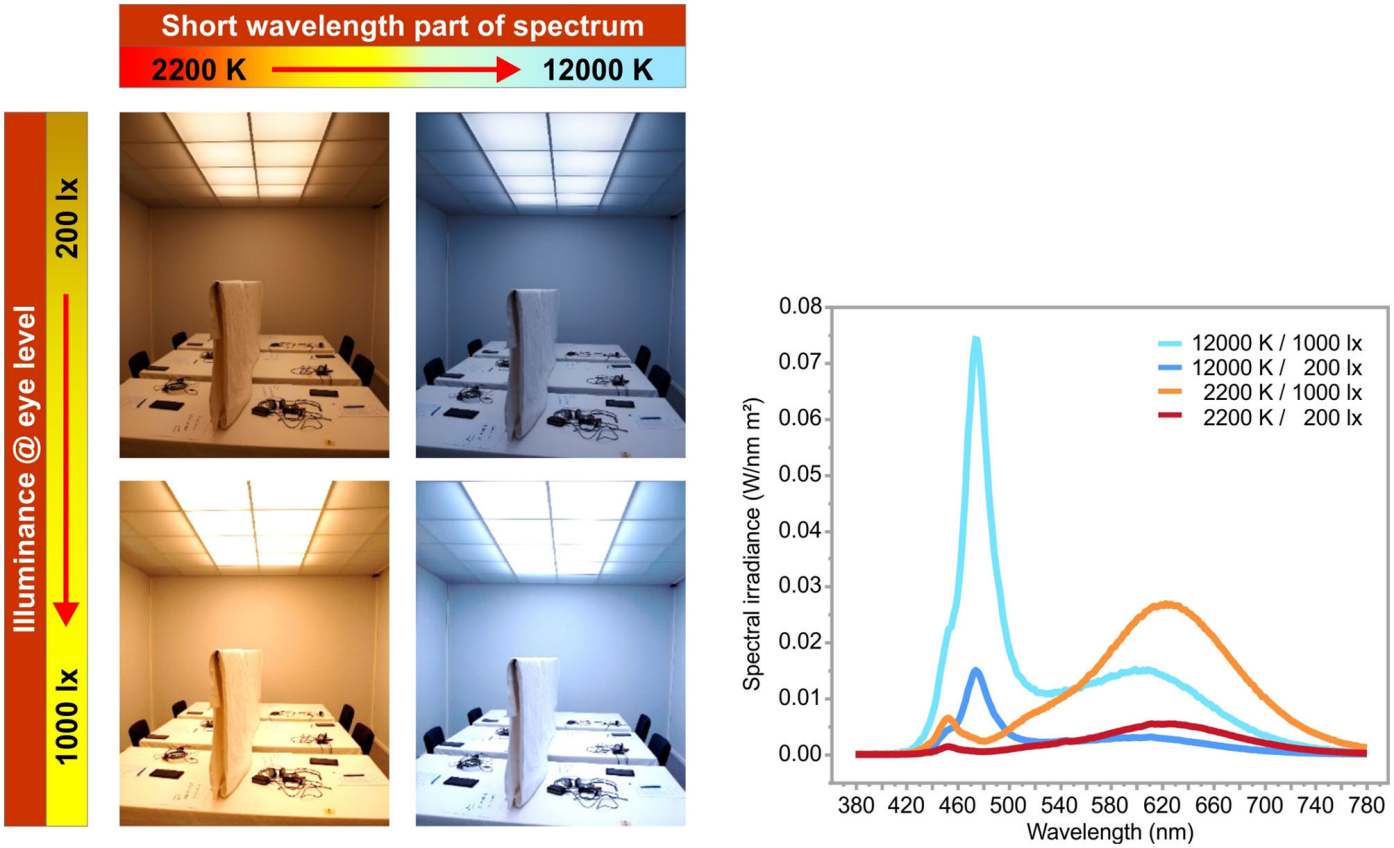
The four lighting conditions **(left)** used in the study with the distribution of their spectral components **(right)**.

One of the subprojects of NiviL focused on human-centric lighting design, i.e., providing always the ideal light conditions for the respective living or working situation, to minimize the adverse effects of artificial lights on mental and physical health in healthy subjects (Cao and Barrionuevo, 2015; Völker, 2018). As part of the NiviL study, two characteristics of light were tested for their influence on alertness: the illuminance (E) at the corneal level of the eye and the short-wavelength content of the spectral power distribution, described by the correlated color temperature (CCT) (Robertson, 1968). Four lighting conditions (Table 4) using two spectral distributions (2,200 K, warm white, and 12,000 K, cool white) and two illuminances (200 lx and 1,000 lx) were tested in a white laboratory without daylight, as shown in Figure 5. Each condition was presented to 30 healthy participants, aged between 18 and 30 years, for 90 min from 9:00 to 10:30 am. To reflect real-life conditions, the amount of participants' sleep during the nights before the study was not regulated. The AuReTim test was conducted twice: after 30 min (#1) and 60 min (#2).

**Table 4:** Lighting parameters of the four tested conditions.

	**Lighting condition**			
	**1**	**2**	**3**	**4**
E_eye_ [lx]	200 ± 20	1,000 ± 100	200 ± 20	1,000 ± 100
Correlated color temperature, CCT [K]	2,200 ± 100	2,200 ± 100	12,000 ± 500	12,000 ± 500
Melanopic action factor^a^, a_mel_	0.3	0.3	1.5	1.5
Melanopic irradiance^b^, E_e, z_ [μW/cm^2^]	9.41	44.71	47.65	236.16
Cyanopic irradianceb, E_e, sc_ [μW/cm^2^]	3.35	15.70	21.41	105.88
Rhodopic irradiance^b^, E_e, r_ [μW/cm^2^]	14.68	70.16	47.52	235.07
Chloropic irradiance^b^, E_e, mc_ [μW/cm^2^]	26.68	128.96	43.05	212.21
Erythropic irradiance^b^, E_e, ic_ [μW/cm^2^]	38.32	186.32	40.02	196.93
Photon density [1/s·cm^2^]	2.5·10^14^	1.2·10^15^	2.5·10^14^	1.3·10^15^
Color rendering index, CRI	87	87	56	56
Horizontal illuminance, E_H_ [lx]	450	1,900	450	1,900

a DIN SPEC 5031-100:2015.

b α-opic irradiance following the SI-compliant approach recommended by CIE (CIE TN 003:2015).

**Figure 6:**
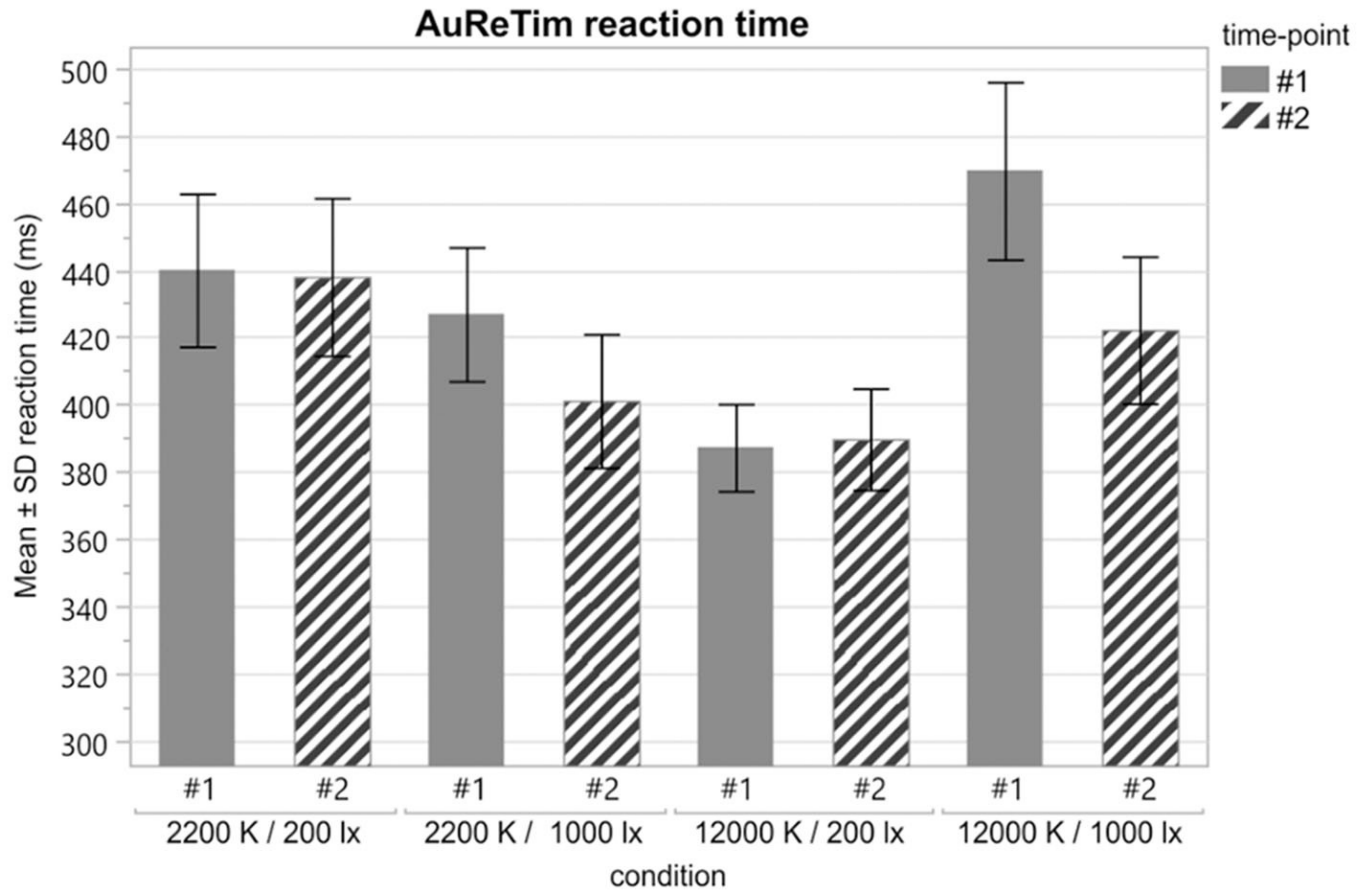
Mean reaction times (error bars indicate ± 1 SD) in milliseconds for each lighting condition at time-point #1 (solid) and time-point #2 (striped).

The reaction time assessed with the AuReTim go/no-go paradigm after spending time under four different lighting conditions (Figure 5, Table 4) showed, that the interaction between illuminance and the spectrum on alertness is more complex than assumed. Short-wavelength light, with a peak wavelength of about 480 nm, is known to be a powerful agent that promotes alertness, performance, and vigilance (Chang et al., 2013) and makes an impact, through photic input to the circadian system, on biological and physiological activities, and health (Cao and Barrionuevo, 2015). However, in this study, high short-wavelength content of the spectrum did not improve alertness in a simple manner. In general, the illuminance level, measured as the corneal level of the eye, was the determining parameter for the effect upon alertness. Cool white light with a correlated color temperature (CCT) of 12,000 K significantly improved the reaction times compared to warm white CCT (2,200 K) of on average 52.8 ms, but only at 200 lx corneal level. 1,000 lx cool white CCT led to longer reaction times of on average 43.6 ms than warm white CCT (Table 5). A further significant interaction was found between time and illuminance (Table 6): For 1,000 lx, reaction times improved between the two test time points #1 and #2, while for 200 lx, reaction times stay constant with time (Table 5, Figure 6) (Rothert, 2020).

**Table 5:** Mean and standard deviations of the reaction times for each lighting condition and time point.

**Lighting condition**		**Reaction time (ms)**			
		**Time-point #1**		**Time-point #2**	
		***N***	**Mean** ±**SD**	***N***	**Mean** ±**SD**
1	2,200 K/200 lx	32	440.0 ± 128.9	32	438.0 ± 132.8
2	2,200 K/1,000 lx	31	426.9 ± 111.0	30	401.1 ± 108.8
3	12,000 K/200 lx	35	387.2 ± 76.6	32	389.7 ± 85.4
4	12,000 K/1,000 lx	28	469.6 ± 139.9	29	422.2 ± 117.9

**Table 6:** Results of the mixed ANOVA test.

	**Effect**	***df***	***F*-value**	***p*-value**	**partial η^2^**
Within subjects	Time	1	6.163	0.014^*^	0.050
	Time × E	1	5.577	0.020^*^	0.045
	Time × CCT	1	0.225	0.636	0.002
Between subjects	Time × E × CCT	1	0.381	0.538	0.003
	E	1	0.757	0.386	0.006
	CCT	1	0.246	0.621	0.002
	E × CCT	1	4.295	0.040^*^	0.035

The ^*^ symbol indicated statistically significant *p*-values, i.e., *p* < 0.05.

Asterisks indicate the level of significance: ^*^p < 0.05, ^**^p < 0.01, ^***^p < 0.001.

## 4. Discussion

Interestingly, the requirements for a PVT have changed little since the introduction of the first portable PVTs by Wilkinson and Houghton: the key idea of a psychomotor vigilance test is measuring the reaction to a briefly presented stimulus, which is widely used in occupational and sleep medicine research. A PVT should be portable and of low weight and size, independent of mains electricity, can store results within the machine, and if necessary operable in absence of the experimenter (Wilkinson and Houghton, 1975). Today, psychomotor vigilance tests are usually administered using personal computer-based software (Khitrov et al., 2014; Reifman et al., 2018) or as a tablet- or smartphone-based portable solutions (Kay et al., 2013; Brunet et al., 2017; Grant et al., 2017). Even though smartphone- and tablet-based PVTs are well accepted by participants and their results are highly correlated to gold-standard PC-based tests, as well as to questionnaires (Kay et al., 2013; Price et al., 2017; Arsintescu et al., 2019), barriers exist especially in elderly or disabled subjects with a reduced capacity for collaboration (Mohadisdudis and Ali, 2014).

AuReTim is a low-cost and simple portable psychomotor vigilance test, which was successfully utilized within several subprojects of NiviL, a joint research project aiming to develop a model for characterizing the non-visual effects of light (Rothert, 2020). Its feasibility was proven in one subproject of NiviL in residents of a retirement home with a mean age of about 85 ± 9 years (standard deviation) (Tübingen, 2019).

AuReTim currently implements two frequently used paradigms for psychomotor vigilance, an unprepared simple reaction test (Wilkinson and Houghton, 1982; Dinges and Powell, 1985; Dorrian et al., 2004), testing the tonic activation of the central nervous system (Weess et al., 2000), and a go/no-go paradigm (Donders, 1969) for testing selective attention (Weess et al., 2000). Both paradigms present auditory stimuli, which the participant is asked to respond to as fast as possible by pressing the hand-held button. Alternatively, the space key of a connected keyboard or the left mouse button of a connected mouse can be used as response input. However, the use of the hand-held button is recommended because the GPIO ports provide a lower and more determinable delay. The latency of GPIO input when using interrupts is about 10–11 μs (Jackowski, 2013). Using Linux as an operating system will add about 50–70 μs (Joan, 2014). The jitter is <1 ms (Beale, 2011). These values are considerably better than those of when using a keyboard or a mouse, which both rely on USB, with a delay in the range of milliseconds.

The simulation experiment showed a high agreement between the expected response time and the response time recorded using the AuReTim software, with limits of agreement between −1.86 and 1.67 ms and only nine of 1,000, and therefore <1% of the recorded responses with more than 4 ms deviation (Table 3). The disparities observed in the range of ~300–400 ms pose a challenge in terms of explanation. One possible factor could be the presence of ongoing initialization processes during or after the software startup. However, it is important to note that the occurrence of such instances is quite infrequent, accounting for only a small proportion in this artificial situation. The wider limits of agreement observed between measured and expected delay (Table 3, Figure 4 center) and between recorded and measured delay (Table 3, Figure 4 right) are most likely attributed to a systematic error stemming from the recording process, specifically the sampling rate (4 ms) of the OpenBCI system, rather than from AuReTim. This assertion is supported by the comparison of delays that have been adjusted using the estimated systematic error associated with the OpenBCI system's sampling rate.

The slight yet statistically significant slope observed in the linear regression analyses between means and differences, suggesting a proportional bias in relation to the response delay, is likely attributed to the OpenBCI system. This bias is evident only in the comparisons involving the measured delay, but not in the comparison between the expected delay and the response delay recorded by AuReTim.

The statistically significant mean difference of −0.10 ms with a standard deviation of <1 ms between the recorded and the expected response delay (Table 3) is well below the maximally allowable margins for timing accuracy of PVT systems of ± 5 ms for bias and 10 ms for the standard deviation as recommended by Basner et al. (2021). The mean difference between the expected and recorded response times of AuReTim is lower compared to other (lab-based) software packages, as documented in the comprehensive timing mega-study conducted by study Bridges et al. (2020). This reduced difference can be attributed to the close integration of the operating system and programming libraries with the Raspberry Pi hardware, a characteristic typically absent in PC-based systems.

AuReTim automatically calculates the response time mean and median as widely used PVT outcome measures (Basner and Dinges, 2011). Additionally, minimum and maximum response time and quartiles as well as the number of total, true-positive, false-negative, and false-positive responses are reported, according to Basner and Dinges (2011). Further measures, like the response speed (Basner and Dinges, 2011), can be calculated from the saved data, which includes each response with timestamps, classification, and response time.

One of the main design goals of AuReTim was extensibility. By leveraging the current input framework, it is easy to implement further stimulus paradigms, for example, an auditory N-Back-Task (Kirchner, 1958), using two different tones similar to the go/no-go paradigm. In addition to auditory stimuli, visual paradigms like the Mackworth Clock-Test (Mackworth, 1948), can easily be added to AuReTim by using either the current touchscreen or by using a larger conventional display connected through HDMI, allowing even combined visual and auditory stimuli, like the Dual N-Back Test (Jaeggi et al., 2003). Recently, an updated version of AuReTim was developed, which now runs on a Raspberry Pi 4 with a 7″ touchscreen and includes, amongst others, the aforementioned visual paradigms as well as several spatial memory update tasks (https://github.com/strator1/AuReTimExtension).

Psychomotor vigilance tests leveraging visual stimulation paradigms may be better suited for tasks requiring fine motor control or visual processing and are amongst the most used measures of sustained attention and highly sensitive to sleepiness caused by, amongst others, sleep deprivation (Jewett et al., 1999; Frey et al., 2004), circadian phase and caffeine administration (Wyatt et al., 2004), administration of melatonin (Graw et al., 2001), and bright light (Wright et al., 1997; Phipps-Nelson et al., 2003). Yet, it is not well investigated if changes in the results of PVT using visual stimuli, e.g., response time or number of lapses, also occur to auditory stimuli. In an extensive study, Jung et al. (2011) compared sustained auditory and visual attention performance assessing multiple metrics of auditory and visual psychomotor vigilance tasks and found auditory vigilance was faster and less variable than visual vigilance. This is in line with results of previous studies that found auditory reaction times to be on average between 19 ms (Jain et al., 2015), 47 ms (Shelton and Kumar, 2010), and up to 71 ms (Solanki et al., 2012) faster than visual reaction times. The difference can be attributed to the time required for a stimulus to reach the brain from the sensory organ: an auditory stimulus takes about 8–10 ms, a visual stimulus about 20–40 ms (Kemp, 1973). This implies that the faster the stimulus reaches the motor cortex, the faster will be the reaction time to the stimulus (Shelton and Kumar, 2010). However, the general pattern of change in attention seems to be similar among sensory–motor behavioral response modalities (Green and Von Gierke, 1984; Jung et al., 2011). In the context of an elder population, auditory stimuli are more suitable than visual stimuli since they bypass visual limitations resulting from age-related visual impairments, such as decreased visual acuity or contrast sensitivity.

AuReTim can be integrated with larger experimental setups, e.g., by using Raspberry Pi interfaces like GPIO along with the Pi4J library: for a different study, the unprepared reaction time to auditory stimuli in different lighting conditions was measured along with the electrical activity of the brain. Different trigger signals (e.g., start of the stimulus, participant response) were sent to an electroencephalogram recording system (actiCHamp, Brain Products GmbH, Gilching, Germany) using a self-made interface between the GPIO ports of the Raspberry Pi and the parallel port (IEEE 1284) interface of the EEG system.

AuReTim is a low-cost and simple portable psychomotor vigilance test, which was successfully utilized within several subprojects of NiviL, a joint research project aiming to develop a model for characterizing the non-visual effects of light. The source code of AuReTim is available under an open-source license (GPLv3) and may be downloaded from https://github.com/strator1/AuReTim. An updated, extended version including, amongst others, visual stimuli is available from https://github.com/strator1/AuReTimExtension. Alternatively, an out-of-the-box version is provided by the STZ eyetrial at the Center for Ophthalmology, Tuebingen, Germany.

## Data availability statement

The original contributions presented in the study are included in the article/Supplementary material, further inquiries can be directed to the corresponding author.

## Ethics statement

Ethical review and approval was not required for the study on human participants in accordance with the local legislation and institutional requirements. The patients/participants provided their written informed consent to participate in this study.

## Author contributions

TS, TH, and TP conceived the presented AuReTim device. TS developed the software and wrote the first version of the manuscript. TH developed the hardware. IR conducted the study and performed the analysis. All authors discussed the results and contributed to the final manuscript.
