# Supplementary material for: AuReTim: an inexpensive and extensible open-source auditory psychomotor vigilance test
Source: Front Sleep. 2023 Jul 21;2:1168209. doi: 10.3389/frsle.2023.1168209 (PMC12713958; doi:10.3389/frsle.2023.1168209)
Supplement: Supplementary file 1 [file Data_Sheet_1.PDF]

Supplementary Material

AuReTim: An inexpensive and extensible open-source auditory psychomotor vigilance test

Torsten Straßer*, Inga Rothert, Thomas Heine, Tobias Peters

*** Correspondence:** Torsten Straßer: torsten.strasser@uni-tuebingen.de

1 Building and installation of AuReTim

AuReTim uses Apache Maven (Massol et al., 2007) as the software project management tool. After installing Maven, the program can be built using the command mvn install in the root directory of the source code. The command will download all required dependencies automatically, build the source code and save the finished packages in the folder “target”.

To install AuReTim on the Raspberry Pi, create the folder “/opt/auretim” and copy the generated JAR archive “AuReTim-VERSION.jar” (whereby “VERSION” is replaced with the current version number of AuReTim), the folder “lib” with its contents, as well as the file “auretim.sh” into it. After copying, grant executable rights to the file “auretim.sh”. Next, install a Java Runtime Environment (JRE) (version ≥ 8) into the folder “/opt/auretim/jre”. Finally, create a user named “auretim” without a password and grant *sudoers*-rights to it. To automatically start AuReTim when booting the Raspberry Pi, append the line “sudo /opt/auretim/auretim.sh” to the file “/etc/rc.local”.

To set up the touchscreen as display and for input, the following files have to be copied to the Raspberry Pi:

- /boot/cmdline.txt
- /boot/config.txt
- /etc/modules

These files are basically required to configure the *ads7846* kernel module used as a driver for the touchscreen display, as well as the settings (e.g. screen orientation) for the display itself. If a different display is used, the configuration may differ.

2 References

Massol, V., van Zyl, J., Porter, B., Casey, J., Sanchez, C., 2007. Better Builds with Maven: How-to Guide for Maven 2.0. DevZuz Library Press.
